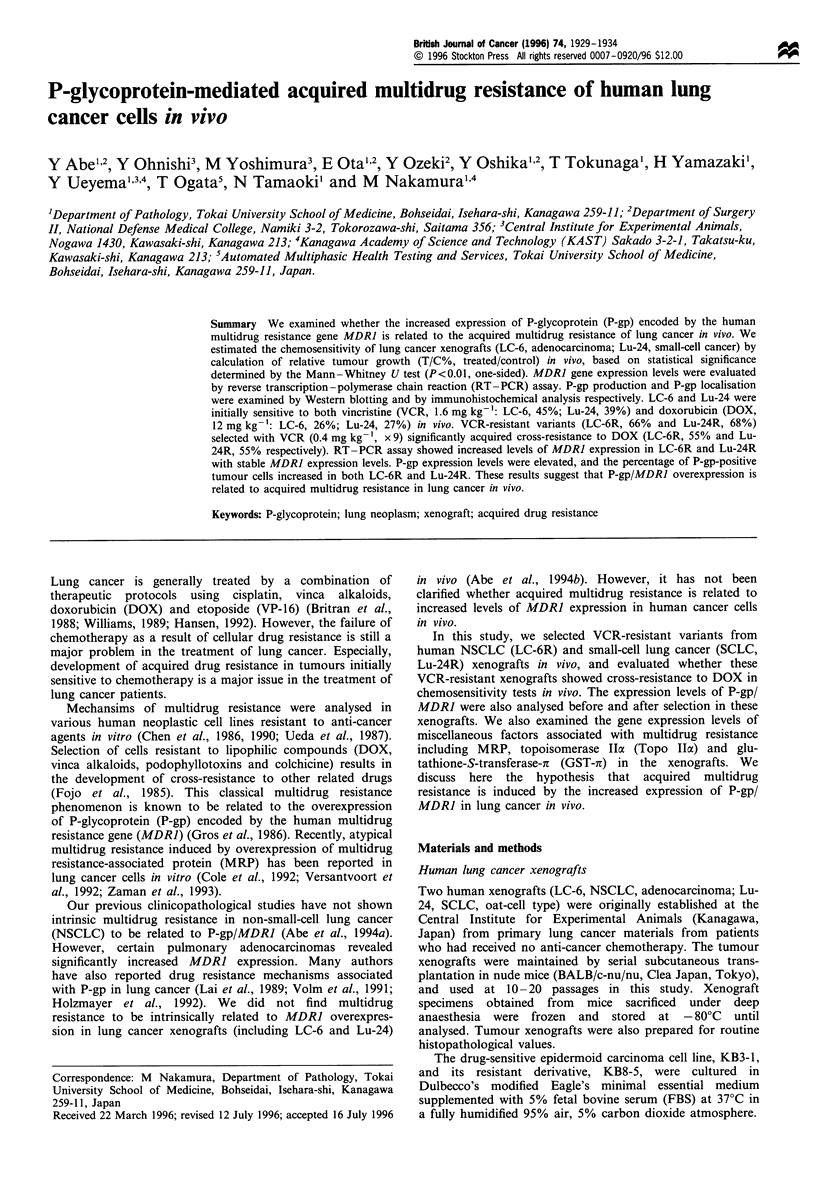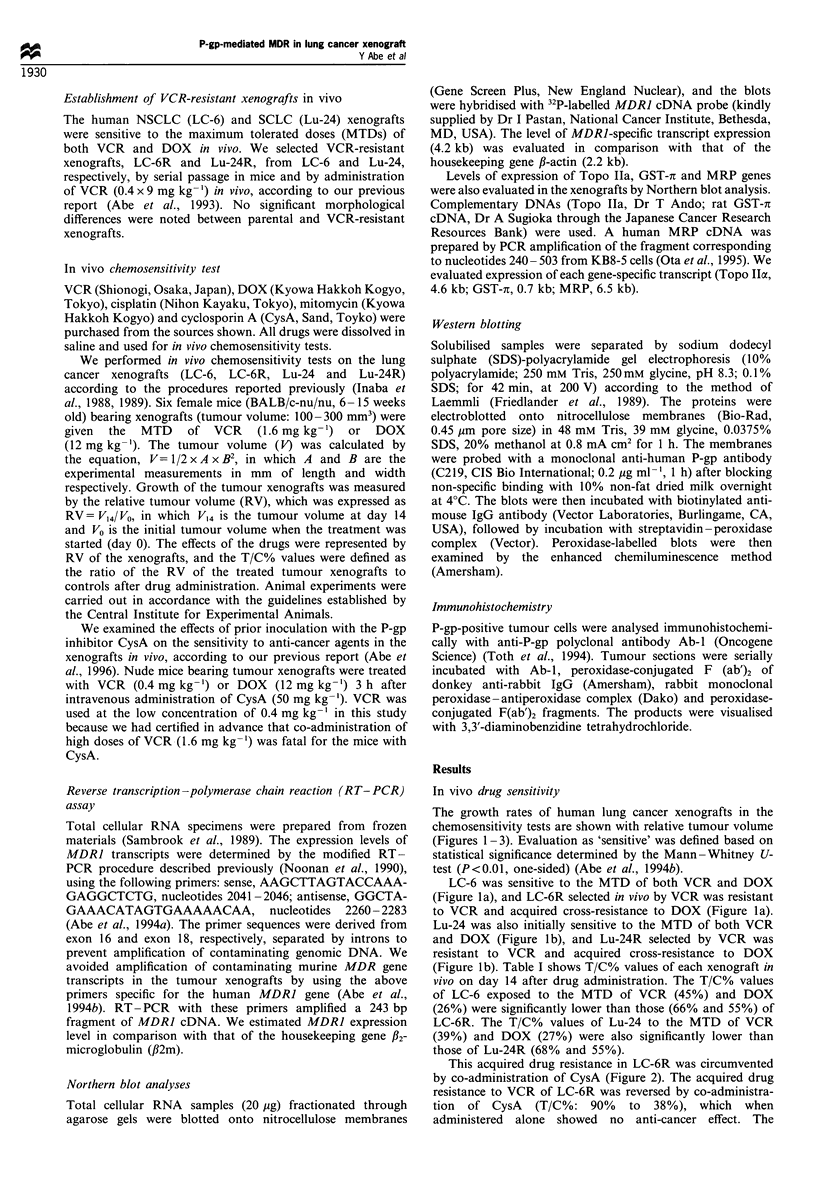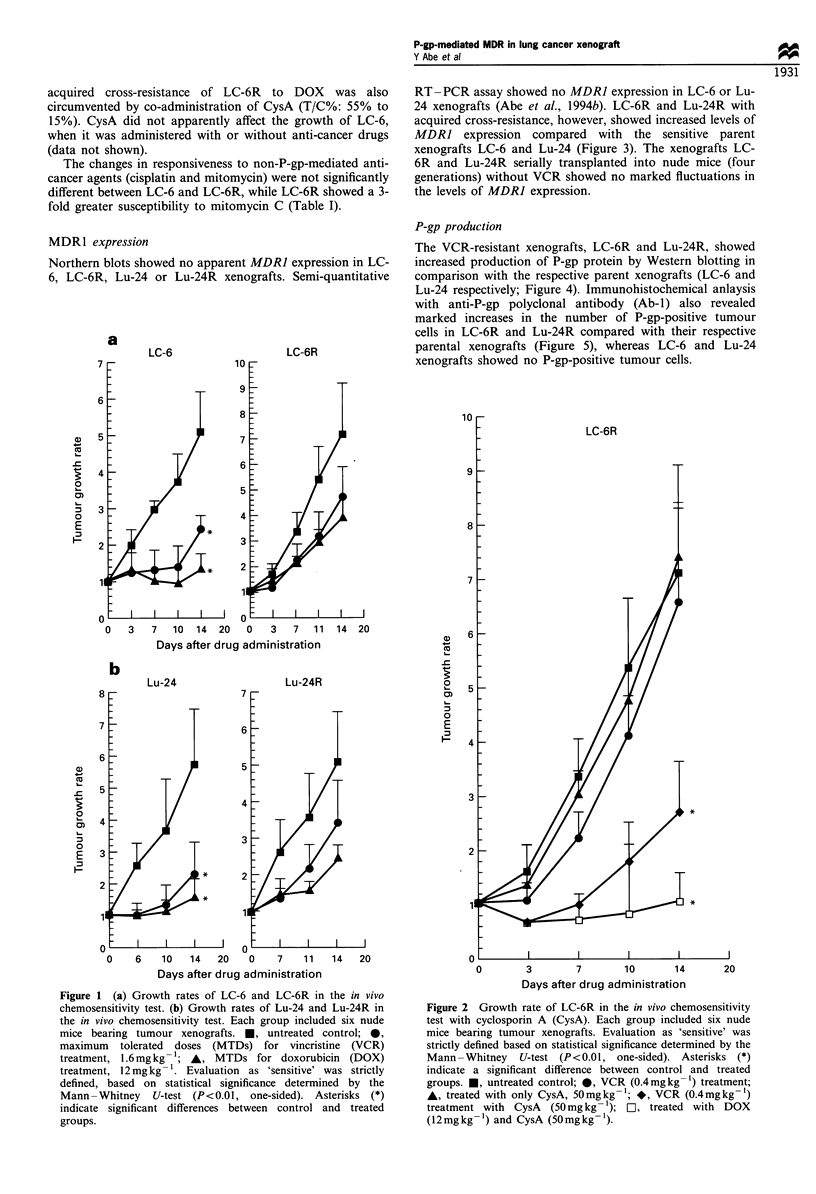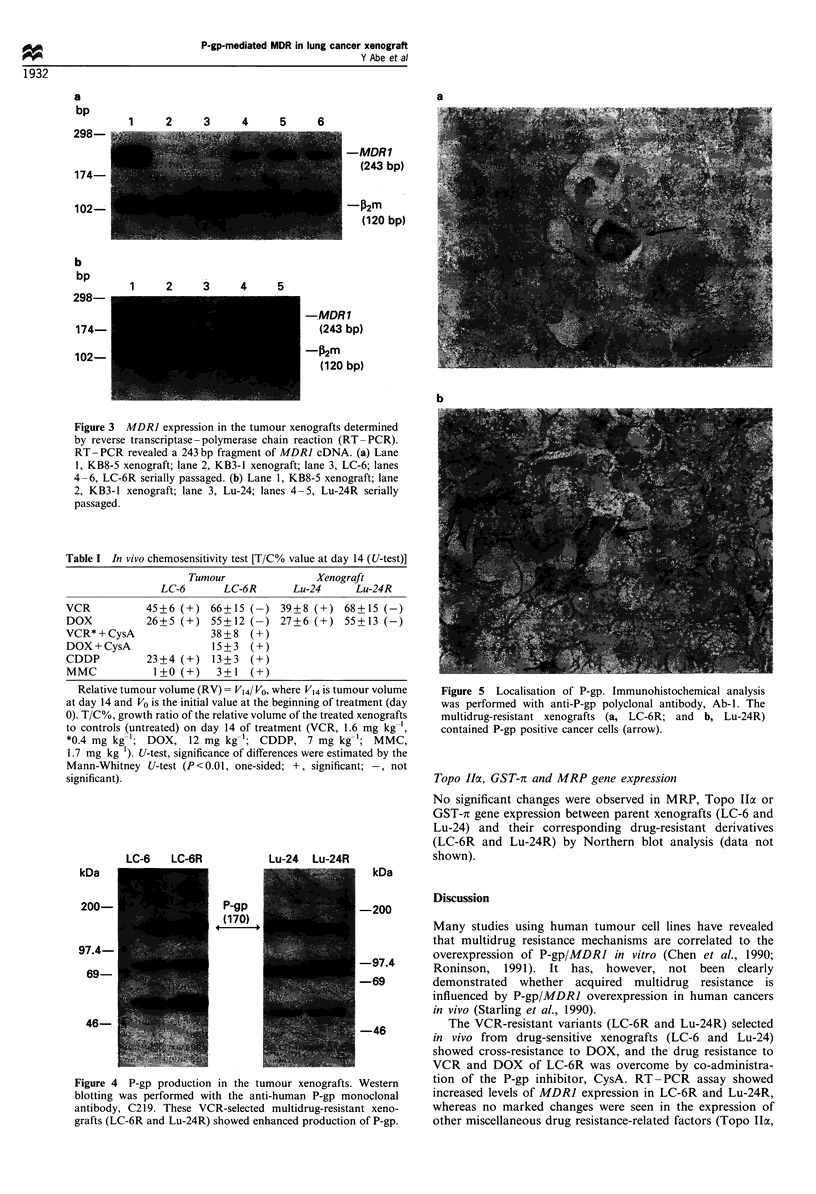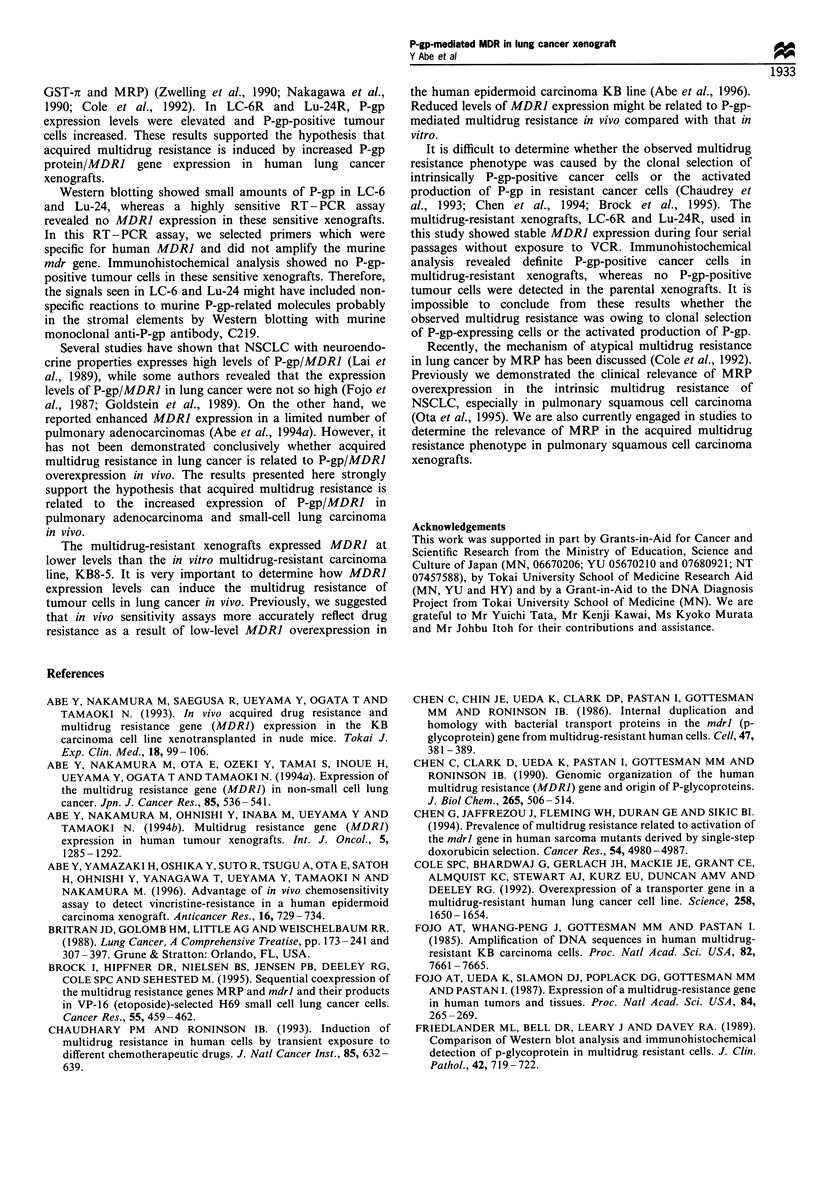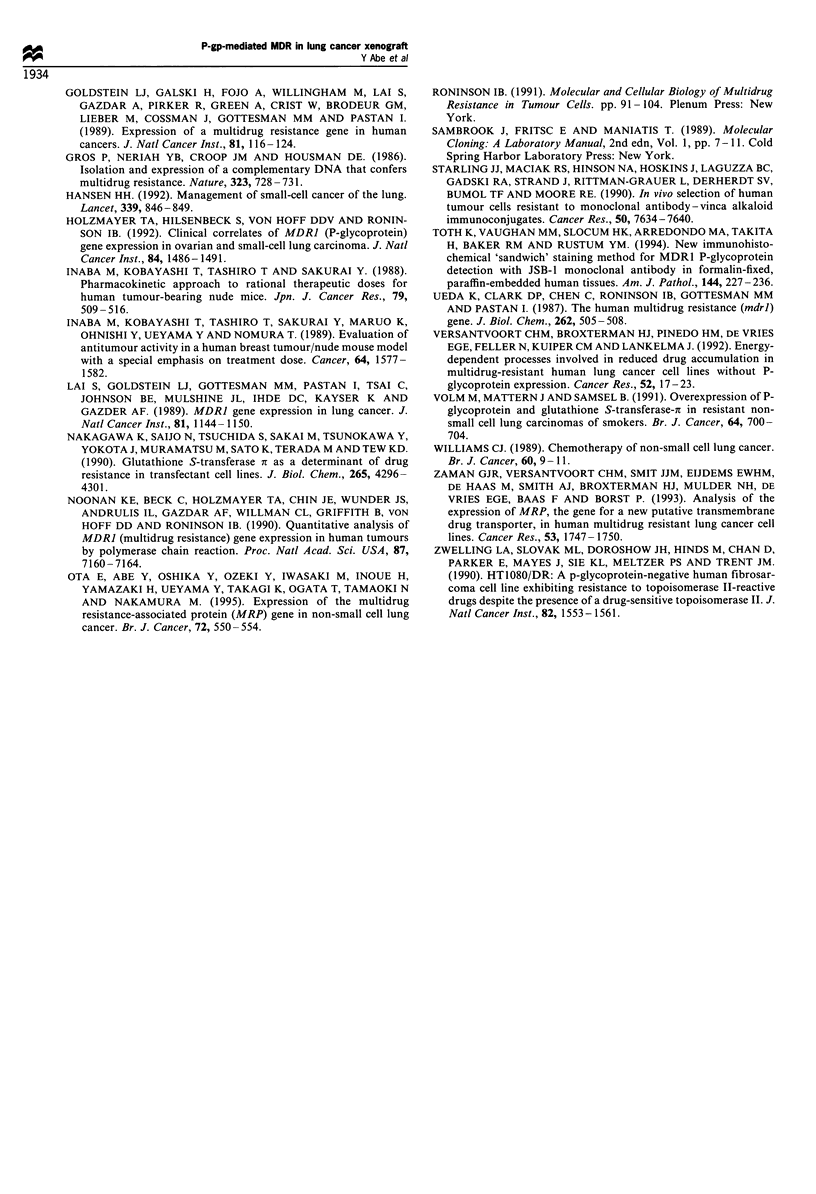# P-glycoprotein-mediated acquired multidrug resistance of human lung cancer cells in vivo.

**DOI:** 10.1038/bjc.1996.655

**Published:** 1996-12

**Authors:** Y. Abe, Y. Ohnishi, M. Yoshimura, E. Ota, Y. Ozeki, Y. Oshika, T. Tokunaga, H. Yamazaki, Y. Ueyema, T. Ogata, N. Tamaoki, M. Nakamura

**Affiliations:** Department of Pathology, Tokai University School of Medicine, Kanagawa, Japan.

## Abstract

**Images:**


British Journal of Cancer (1996) 74, 1929-1934
© 1996 Stockton Press All rights reserved 0007-0920/96 $12.00

### P-glycoprotein-mediated acquired multidrug resistance of human lung cancer cells *in vivo*

Y Abe[1,2], Y Ohnishi[3], M Yoshimura[3], E Ota[1,2], Y Ozeki[2], Y Oshika[1,2], T Tokunaga[1], H Yamazaki[1], Y Ueyema[1,3,4], T Ogata[5], N Tamaoki[1] and M Nakamura[1,4]

[1]*Department of Pathology, Tokai University School of Medicine, Bohseidai, Isehara-shi, Kanagawa 259-11;* [2]*Department of Surgery II, National Defense Medical College, Namiki 3-2, Tokorozawa-shi, Saitama 356;* [3]*Central Institute for Experimental Animals, Nogawa 1430, Kawasaki-shi, Kanagawa 213;* [4]*Kanagawa Academy of Science and Technology (KAST) Sakado 3-2-1, Takatsu-ku, Kawasaki-shi, Kanagawa 213;* [5]*Automated Multiphasic Health Testing and Services, Tokai University School of Medicine, Bohseidai, Isehara-shi, Kanagawa 259-11, Japan.*

**Summary** We examined whether the increased expression of P-glycoprotein (P-gp) encoded by the human multidrug resistance gene *MDR1* is related to the acquired multidrug resistance of lung cancer *in vivo*. We estimated the chemosensitivity of lung cancer xenografts (LC-6, adenocarcinoma; Lu-24, small-cell cancer) by calculation of relative tumour growth (T/C%, treated/control) *in vivo*, based on statistical significance determined by the Mann-Whitney *U* test ($P<0.01$, one-sided). *MDR1* gene expression levels were evaluated by reverse transcription-polymerase chain reaction (RT-PCR) assay. P-gp production and P-gp localisation were examined by Western blotting and by immunohistochemical analysis respectively. LC-6 and Lu-24 were initially sensitive to both vincristine (VCR, 1.6 mg kg$^{-1}$: LC-6, 45%; Lu-24, 39%) and doxorubicin (DOX, 12 mg kg$^{-1}$: LC-6, 26%; Lu-24, 27%) *in vivo*. VCR-resistant variants (LC-6R, 66% and Lu-24R, 68%) selected with VCR (0.4 mg kg$^{-1}$, ×9) significantly acquired cross-resistance to DOX (LC-6R, 55% and Lu-24R, 55% respectively). RT-PCR assay showed increased levels of *MDR1* expression in LC-6R and Lu-24R with stable *MDR1* expression levels. P-gp expression levels were elevated, and the percentage of P-gp-positive tumour cells increased in both LC-6R and Lu-24R. These results suggest that P-gp/*MDR1* overexpression is related to acquired multidrug resistance in lung cancer *in vivo*.

**Keywords:** P-glycoprotein; lung neoplasm; xenograft; acquired drug resistance

Lung cancer is generally treated by a combination of therapeutic protocols using cisplatin, vinca alkaloids, doxorubicin (DOX) and etoposide (VP-16) (Britran *et al.*, 1988; Williams, 1989; Hansen, 1992). However, the failure of chemotherapy as a result of cellular drug resistance is still a major problem in the treatment of lung cancer. Especially, development of acquired drug resistance in tumours initially sensitive to chemotherapy is a major issue in the treatment of lung cancer patients.

Mechansims of multidrug resistance were analysed in various human neoplastic cell lines resistant to anti-cancer agents *in vitro* (Chen *et al.*, 1986, 1990; Ueda *et al.*, 1987). Selection of cells resistant to lipophilic compounds (DOX, vinca alkaloids, podophyllotoxins and colchicine) results in the development of cross-resistance to other related drugs (Fojo *et al.*, 1985). This classical multidrug resistance phenomenon is known to be related to the overexpression of P-glycoprotein (P-gp) encoded by the human multidrug resistance gene (*MDR1*) (Gros *et al.*, 1986). Recently, atypical multidrug resistance induced by overexpression of multidrug resistance-associated protein (MRP) has been reported in lung cancer cells *in vitro* (Cole *et al.*, 1992; Versantvoort *et al.*, 1992; Zaman *et al.*, 1993).

Our previous clinicopathological studies have not shown intrinsic multidrug resistance in non-small-cell lung cancer (NSCLC) to be related to P-gp/*MDR1* (Abe *et al.*, 1994*a*). However, certain pulmonary adenocarcinomas revealed significantly increased *MDR1* expression. Many authors have also reported drug resistance mechanisms associated with P-gp in lung cancer (Lai *et al.*, 1989; Volm *et al.*, 1991; Holzmayer *et al.*, 1992). We did not find multidrug resistance to be intrinsically related to *MDR1* overexpression in lung cancer xenografts (including LC-6 and Lu-24) *in vivo* (Abe *et al.*, 1994*b*). However, it has not been clarified whether acquired multidrug resistance is related to increased levels of *MDR1* expression in human cancer cells *in vivo*.

In this study, we selected VCR-resistant variants from human NSCLC (LC-6R) and small-cell lung cancer (SCLC, Lu-24R) xenografts *in vivo*, and evaluated whether these VCR-resistant xenografts showed cross-resistance to DOX in chemosensitivity tests *in vivo*. The expression levels of P-gp/*MDR1* were also analysed before and after selection in these xenografts. We also examined the gene expression levels of miscellaneous factors associated with multidrug resistance including MRP, topoisomerase IIα (Topo IIα) and glutathione-*S*-transferase-π (GST-π) in the xenografts. We discuss here the hypothesis that acquired multidrug resistance is induced by the increased expression of P-gp/*MDR1* in lung cancer *in vivo*.

### Materials and methods

### *Human lung cancer xenografts*

Two human xenografts (LC-6, NSCLC, adenocarcinoma; Lu-24, SCLC, oat-cell type) were originally established at the Central Institute for Experimental Animals (Kanagawa, Japan) from primary lung cancer materials from patients who had received no anti-cancer chemotherapy. The tumour xenografts were maintained by serial subcutaneous transplantation in nude mice (BALB/c-nu/nu, Clea Japan, Tokyo), and used at 10-20 passages in this study. Xenograft specimens obtained from mice sacrificed under deep anaesthesia were frozen and stored at −80°C until analysed. Tumour xenografts were also prepared for routine histopathological values.

The drug-sensitive epidermoid carcinoma cell line, KB3-1, and its resistant derivative, KB8-5, were cultured in Dulbecco's modified Eagle's minimal essential medium supplemented with 5% fetal bovine serum (FBS) at 37°C in a fully humidified 95% air, 5% carbon dioxide atmosphere.

Correspondence: M Nakamura, Department of Pathology, Tokai University School of Medicine, Bohseidai, Isehara-shi, Kanagawa 259-11, Japan
Received 22 March 1996; revised 12 July 1996; accepted 16 July 1996

*Establishment of VCR-resistant xenografts* in vivo

The human NSCLC (LC-6) and SCLC (Lu-24) xenografts were sensitive to the maximum tolerated doses (MTDs) of both VCR and DOX *in vivo*. We selected VCR-resistant xenografts, LC-6R and Lu-24R, from LC-6 and Lu-24, respectively, by serial passage in mice and by administration of VCR ($0.4 \times 9$ mg $kg^{-1}$) *in vivo*, according to our previous report (Abe *et al.*, 1993). No significant morphological differences were noted between parental and VCR-resistant xenografts.

In vivo *chemosensitivity test*

VCR (Shionogi, Osaka, Japan), DOX (Kyowa Hakkoh Kogyo, Tokyo), cisplatin (Nihon Kayaku, Tokyo), mitomycin (Kyowa Hakkoh Kogyo) and cyclosporin A (CysA, Sand, Toyko) were purchased from the sources shown. All drugs were dissolved in saline and used for *in vivo* chemosensitivity tests.

We performed *in vivo* chemosensitivity tests on the lung cancer xenografts (LC-6, LC-6R, Lu-24 and Lu-24R) according to the procedures reported previously (Inaba *et al.*, 1988, 1989). Six female mice (BALB/c-nu/nu, 6–15 weeks old) bearing xenografts (tumour volume: 100–300 $mm^3$) were given the MTD of VCR (1.6 mg $kg^{-1}$) or DOX (12 mg $kg^{-1}$). The tumour volume ($V$) was calculated by the equation, $V = 1/2 \times A \times B^2$, in which $A$ and $B$ are the experimental measurements in mm of length and width respectively. Growth of the tumour xenografts was measured by the relative tumour volume (RV), which was expressed as $RV = V_{14}/V_0$, in which $V_{14}$ is the tumour volume at day 14 and $V_0$ is the initial tumour volume when the treatment was started (day 0). The effects of the drugs were represented by RV of the xenografts, and the T/C% values were defined as the ratio of the RV of the treated tumour xenografts to controls after drug administration. Animal experiments were carried out in accordance with the guidelines established by the Central Institute for Experimental Animals.

We examined the effects of prior inoculation with the P-gp inhibitor CysA on the sensitivity to anti-cancer agents in the xenografts *in vivo*, according to our previous report (Abe *et al.*, 1996). Nude mice bearing tumour xenografts were treated with VCR (0.4 mg $kg^{-1}$) or DOX (12 mg $kg^{-1}$) 3 h after intravenous administration of CysA (50 mg $kg^{-1}$). VCR was used at the low concentration of 0.4 mg $kg^{-1}$ in this study because we had certified in advance that co-administration of high doses of VCR (1.6 mg $kg^{-1}$) was fatal for the mice with CysA.

*Reverse transcription–polymerase chain reaction (RT–PCR) assay*

Total cellular RNA specimens were prepared from frozen materials (Sambrook *et al.*, 1989). The expression levels of *MDR1* transcripts were determined by the modified RT–PCR procedure described previously (Noonan *et al.*, 1990), using the following primers: sense, AAGCTTAGTACCAAA-GAGGCTCTG, nucleotides 2041–2046; antisense, GGCTA-GAAACATAGTGAAAAACAA, nucleotides 2260–2283 (Abe *et al.*, 1994*a*). The primer sequences were derived from exon 16 and exon 18, respectively, separated by introns to prevent amplification of contaminating genomic DNA. We avoided amplification of contaminating murine *MDR* gene transcripts in the tumour xenografts by using the above primers specific for the human *MDR1* gene (Abe *et al.*, 1994*b*). RT–PCR with these primers amplified a 243 bp fragment of *MDR1* cDNA. We estimated *MDR1* expression level in comparison with that of the housekeeping gene $\beta_2$-microglobulin ($\beta$2m).

*Northern blot analyses*

Total cellular RNA samples (20 $\mu$g) fractionated through agarose gels were blotted onto nitrocellulose membranes (Gene Screen Plus, New England Nuclear), and the blots were hybridised with $^{32}$P-labelled *MDR1* cDNA probe (kindly supplied by Dr I Pastan, National Cancer Institute, Bethesda, MD, USA). The level of *MDR1*-specific transcript expression (4.2 kb) was evaluated in comparison with that of the housekeeping gene $\beta$-actin (2.2 kb).

Levels of expression of Topo IIa, GST-$\pi$ and MRP genes were also evaluated in the xenografts by Northern blot analysis. Complementary DNAs (Topo IIa, Dr T Ando; rat GST-$\pi$ cDNA, Dr A Sugioka through the Japanese Cancer Research Resources Bank) were used. A human MRP cDNA was prepared by PCR amplification of the fragment corresponding to nucleotides 240–503 from KB8-5 cells (Ota *et al.*, 1995). We evaluated expression of each gene-specific transcript (Topo II$\alpha$, 4.6 kb; GST-$\pi$, 0.7 kb; MRP, 6.5 kb).

*Western blotting*

Solubilised samples were separated by sodium dodecyl sulphate (SDS)-polyacrylamide gel electrophoresis (10% polyacrylamide; 250 mM Tris, 250 mM glycine, pH 8.3; 0.1% SDS; for 42 min, at 200 V) according to the method of Laemmli (Friedlander *et al.*, 1989). The proteins were electroblotted onto nitrocellulose membranes (Bio-Rad, 0.45 $\mu$m pore size) in 48 mM Tris, 39 mM glycine, 0.0375% SDS, 20% methanol at 0.8 mA $cm^2$ for 1 h. The membranes were probed with a monoclonal anti-human P-gp antibody (C219, CIS Bio International; 0.2 $\mu$g $ml^{-1}$, 1 h) after blocking non-specific binding with 10% non-fat dried milk overnight at 4°C. The blots were then incubated with biotinylated anti-mouse IgG antibody (Vector Laboratories, Burlingame, CA, USA), followed by incubation with streptavidin–peroxidase complex (Vector). Peroxidase-labelled blots were then examined by the enhanced chemiluminescence method (Amersham).

*Immunohistochemistry*

P-gp-positive tumour cells were analysed immunohistochemically with anti-P-gp polyclonal antibody Ab-1 (Oncogene Science) (Toth *et al.*, 1994). Tumour sections were serially incubated with Ab-1, peroxidase-conjugated F $(ab')_2$ of donkey anti-rabbit IgG (Amersham), rabbit monoclonal peroxidase–antiperoxidase complex (Dako) and peroxidase-conjugated $F(ab')_2$ fragments. The products were visualised with 3,3′-diaminobenzidine tetrahydrochloride.

### Results

In vivo *drug sensitivity*

The growth rates of human lung cancer xenografts in the chemosensitivity tests are shown with relative tumour volume (Figures 1–3). Evaluation as 'sensitive' was defined based on statistical significance determined by the Mann–Whitney *U*-test ($P < 0.01$, one-sided) (Abe *et al.*, 1994*b*).

LC-6 was sensitive to the MTD of both VCR and DOX (Figure 1a), and LC-6R selected *in vivo* by VCR was resistant to VCR and acquired cross-resistance to DOX (Figure 1a). Lu-24 was also initially sensitive to the MTD of both VCR and DOX (Figure 1b), and Lu-24R selected by VCR was resistant to VCR and acquired cross-resistance to DOX (Figure 1b). Table I shows T/C% values of each xenograft *in vivo* on day 14 after drug administration. The T/C% values of LC-6 exposed to the MTD of VCR (45%) and DOX (26%) were significantly lower than those (66% and 55%) of LC-6R. The T/C% values of Lu-24 to the MTD of VCR (39%) and DOX (27%) were also significantly lower than those of Lu-24R (68% and 55%).

This acquired drug resistance in LC-6R was circumvented by co-administration of CysA (Figure 2). The acquired drug resistance to VCR of LC-6R was reversed by co-administration of CysA (T/C%: 90% to 38%), which when administered alone showed no anti-cancer effect. The acquired cross-resistance of LC-6R to DOX was also circumvented by co-administration of CysA (T/C%: 55% to 15%). CysA did not apparently affect the growth of LC-6, when it was administered with or without anti-cancer drugs (data not shown).

The changes in responsiveness to non-P-gp-mediated anti-cancer agents (cisplatin and mitomycin) were not significantly different between LC-6 and LC-6R, while LC-6R showed a 3-fold greater susceptibility to mitomycin C (Table I).

### MDR1 *expression*

Northern blots showed no apparent *MDR1* expression in LC-6, LC-6R, Lu-24 or Lu-24R xenografts. Semi-quantitative RT-PCR assay showed no *MDR1* expression in LC-6 or Lu-24 xenografts (Abe *et al.*, 1994*b*). LC-6R and Lu-24R with acquired cross-resistance, however, showed increased levels of *MDR1* expression compared with the sensitive parent xenografts LC-6 and Lu-24 (Figure 3). The xenografts LC-6R and Lu-24R serially transplanted into nude mice (four generations) without VCR showed no marked fluctuations in the levels of *MDR1* expression.

### *P-gp production*

The VCR-resistant xenografts, LC-6R and Lu-24R, showed increased production of P-gp protein by Western blotting in comparison with the respective parent xenografts (LC-6 and Lu-24 respectively; Figure 4). Immunohistochemical anlaysis with anti-P-gp polyclonal antibody (Ab-1) also revealed marked increases in the number of P-gp-positive tumour cells in LC-6R and Lu-24R compared with their respective parental xenografts (Figure 5), whereas LC-6 and Lu-24 xenografts showed no P-gp-positive tumour cells.

**Figure 1** (**a**) Growth rates of LC-6 and LC-6R in the *in vivo* chemosensitivity test. (**b**) Growth rates of Lu-24 and Lu-24R in the *in vivo* chemosensitivity test. Each group included six nude mice bearing tumour xenografts. ■, untreated control; ●, maximum tolerated doses (MTDs) for vincristine (VCR) treatment, $1.6\,mg\,kg^{-1}$; ▲, MTDs for doxorubicin (DOX) treatment, $12\,mg\,kg^{-1}$. Evaluation as 'sensitive' was strictly defined, based on statistical significance determined by the Mann-Whitney *U*-test ($P<0.01$, one-sided). Asterisks (*) indicate significant differences between control and treated groups.

**Figure 2** Growth rate of LC-6R in the *in vivo* chemosensitivity test with cyclosporin A (CysA). Each group included six nude mice bearing tumour xenografts. Evaluation as 'sensitive' was strictly defined based on statistical significance determined by the Mann-Whitney *U*-test ($P<0.01$, one-sided). Asterisks (*) indicate a significant difference between control and treated groups. ■, untreated control; ●, VCR ($0.4\,mg\,kg^{-1}$) treatment; ▲, treated with only CysA, $50\,mg\,kg^{-1}$; ◆, VCR ($0.4\,mg\,kg^{-1}$) treatment with CysA ($50\,mg\,kg^{-1}$); □, treated with DOX ($12\,mg\,kg^{-1}$) and CysA ($50\,mg\,kg^{-1}$).

**Figure 3** *MDR1* expression in the tumour xenografts determined by reverse transcriptase–polymerase chain reaction (RT–PCR). RT–PCR revealed a 243 bp fragment of *MDR1* cDNA. (**a**) Lane 1, KB8-5 xenograft; lane 2, KB3-1 xenograft; lane 3, LC-6; lanes 4–6, LC-6R serially passaged. (**b**) Lane 1, KB8-5 xenograft; lane 2, KB3-1 xenograft; lane 3, Lu-24; lanes 4–5, Lu-24R serially passaged.

**Table I** *In vivo* chemosensitivity test [T/C% value at day 14 (*U*-test)]

| | *Tumour* | | *Xenograft* | |
|---|---|---|---|---|
| | *LC-6* | *LC-6R* | *Lu-24* | *Lu-24R* |
| VCR | 45±6 (+) | 66±15 (−) | 39±8 (+) | 68±15 (−) |
| DOX | 26±5 (+) | 55±12 (−) | 27±6 (+) | 55±13 (−) |
| VCR* + CysA | | 38±8 (+) | | |
| DOX + CysA | | 15±3 (+) | | |
| CDDP | 23±4 (+) | 13±3 (+) | | |
| MMC | 1±0 (+) | 3±1 (+) | | |

Relative tumour volume (RV) = $V_{14}/V_0$, where $V_{14}$ is tumour volume at day 14 and $V_0$ is the initial value at the beginning of treatment (day 0). T/C%, growth ratio of the relative volume of the treated xenografts to controls (untreated) on day 14 of treatment (VCR, 1.6 mg $kg^{-1}$, *0.4 mg $kg^{-1}$; DOX, 12 mg $kg^{-1}$; CDDP, 7 mg $kg^{-1}$; MMC, 1.7 mg $kg^{-1}$). *U*-test, significance of differences were estimated by the Mann-Whitney *U*-test ($P<0.01$, one-sided; +, significant; −, not significant).

**Figure 4** P-gp production in the tumour xenografts. Western blotting was performed with the anti-human P-gp monoclonal antibody, C219. These VCR-selected multidrug-resistant xenografts (LC-6R and Lu-24R) showed enhanced production of P-gp.

**Figure 5** Localisation of P-gp. Immunohistochemical analysis was performed with anti-P-gp polyclonal antibody, Ab-1. The multidrug-resistant xenografts (**a**, LC-6R; and **b**, Lu-24R) contained P-gp positive cancer cells (arrow).

*Topo IIα, GST-π and MRP gene expression*

No significant changes were observed in MRP, Topo IIα or GST-π gene expression between parent xenografts (LC-6 and Lu-24) and their corresponding drug-resistant derivatives (LC-6R and Lu-24R) by Northern blot analysis (data not shown).

### Discussion

Many studies using human tumour cell lines have revealed that multidrug resistance mechanisms are correlated to the overexpression of P-gp/*MDR1 in vitro* (Chen *et al.*, 1990; Roninson, 1991). It has, however, not been clearly demonstrated whether acquired multidrug resistance is influenced by P-gp/*MDR1* overexpression in human cancers *in vivo* (Starling *et al.*, 1990).

The VCR-resistant variants (LC-6R and Lu-24R) selected *in vivo* from drug-sensitive xenografts (LC-6 and Lu-24) showed cross-resistance to DOX, and the drug resistance to VCR and DOX of LC-6R was overcome by co-administration of the P-gp inhibitor, CysA. RT–PCR assay showed increased levels of *MDR1* expression in LC-6R and Lu-24R, whereas no marked changes were seen in the expression of other miscellaneous drug resistance-related factors (Topo IIα,

GST-$\pi$ and MRP) (Zwelling *et al.*, 1990; Nakagawa *et al.*, 1990; Cole *et al.*, 1992). In LC-6R and Lu-24R, P-gp expression levels were elevated and P-gp-positive tumour cells increased. These results supported the hypothesis that acquired multidrug resistance is induced by increased P-gp protein/*MDR1* gene expression in human lung cancer xenografts.

Western blotting showed small amounts of P-gp in LC-6 and Lu-24, whereas a highly sensitive RT–PCR assay revealed no *MDR1* expression in these sensitive xenografts. In this RT–PCR assay, we selected primers which were specific for human *MDR1* and did not amplify the murine *mdr* gene. Immunohistochemical analysis showed no P-gp-positive tumour cells in these sensitive xenografts. Therefore, the signals seen in LC-6 and Lu-24 might have included non-specific reactions to murine P-gp-related molecules probably in the stromal elements by Western blotting with murine monoclonal anti-P-gp antibody, C219.

Several studies have shown that NSCLC with neuroendocrine properties expresses high levels of P-gp/*MDR1* (Lai *et al.*, 1989), while some authors revealed that the expression levels of P-gp/*MDR1* in lung cancer were not so high (Fojo *et al.*, 1987; Goldstein *et al.*, 1989). On the other hand, we reported enhanced *MDR1* expression in a limited number of pulmonary adenocarcinomas (Abe *et al.*, 1994*a*). However, it has not been demonstrated conclusively whether acquired multidrug resistance in lung cancer is related to P-gp/*MDR1* overexpression *in vivo*. The results presented here strongly support the hypothesis that acquired multidrug resistance is related to the increased expression of P-gp/*MDR1* in pulmonary adenocarcinoma and small-cell lung carcinoma *in vivo*.

The multidrug-resistant xenografts expressed *MDR1* at lower levels than the *in vitro* multidrug-resistant carcinoma line, KB8-5. It is very important to determine how *MDR1* expression levels can induce the multidrug resistance of tumour cells in lung cancer *in vivo*. Previously, we suggested that *in vivo* sensitivity assays more accurately reflect drug resistance as a result of low-level *MDR1* overexpression in the human epidermoid carcinoma KB line (Abe *et al.*, 1996). Reduced levels of *MDR1* expression might be related to P-gp-mediated multidrug resistance *in vivo* compared with that *in vitro*.

It is difficult to determine whether the observed multidrug resistance phenotype was caused by the clonal selection of intrinsically P-gp-positive cancer cells or the activated production of P-gp in resistant cancer cells (Chaudrey *et al.*, 1993; Chen *et al.*, 1994; Brock *et al.*, 1995). The multidrug-resistant xenografts, LC-6R and Lu-24R, used in this study showed stable *MDR1* expression during four serial passages without exposure to VCR. Immunohistochemical analysis revealed definite P-gp-positive cancer cells in multidrug-resistant xenografts, whereas no P-gp-positive tumour cells were detected in the parental xenografts. It is impossible to conclude from these results whether the observed multidrug resistance was owing to clonal selection of P-gp-expressing cells or the activated production of P-gp.

Recently, the mechanism of atypical multidrug resistance in lung cancer by MRP has been discussed (Cole *et al.*, 1992). Previously we demonstrated the clinical relevance of MRP overexpression in the intrinsic multidrug resistance of NSCLC, especially in pulmonary squamous cell carcinoma (Ota *et al.*, 1995). We are also currently engaged in studies to determine the relevance of MRP in the acquired multidrug resistance phenotype in pulmonary squamous cell carcinoma xenografts.

**Acknowledgements**
This work was supported in part by Grants-in-Aid for Cancer and Scientific Research from the Ministry of Education, Science and Culture of Japan (MN, 06670206; YU 05670210 and 07680921; NT 07457588), by Tokai University School of Medicine Research Aid (MN, YU and HY) and by a Grant-in-Aid to the DNA Diagnosis Project from Tokai University School of Medicine (MN). We are grateful to Mr Yuichi Tata, Mr Kenji Kawai, Ms Kyoko Murata and Mr Johbu Itoh for their contributions and assistance.

### References

ABE Y, NAKAMURA M, SAEGUSA R, UEYAMA Y, OGATA T AND TAMAOKI N. (1993). *In vivo* acquired drug resistance and multidrug resistance gene (*MDR1*) expression in the KB carcinoma cell line xenotransplanted in nude mice. *Tokai J. Exp. Clin. Med.*, **18**, 99–106.

ABE Y, NAKAMURA M, OTA E, OZEKI Y, TAMAI S, INOUE H, UEYAMA Y, OGATA T AND TAMAOKI N. (1994*a*). Expression of the multidrug resistance gene (*MDR1*) in non-small cell lung cancer. *Jpn. J. Cancer Res.*, **85**, 536–541.

ABE Y, NAKAMURA M, OHNISHI Y, INABA M, UEYAMA Y AND TAMAOKI N. (1994*b*). Multidrug resistance gene (*MDR1*) expression in human tumour xenografts. *Int. J. Oncol.*, **5**, 1285–1292.

ABE Y, YAMAZAKI H, OSHIKA Y, SUTO R, TSUGU A, OTA E, SATOH H, OHNISHI Y, YANAGAWA T, UEYAMA Y, TAMAOKI N AND NAKAMURA M. (1996). Advantage of *in vivo* chemosensitivity assay to detect vincristine-resistance in a human epidermoid carcinoma xenograft. *Anticancer Res.*, **16**, 729–734.

BRITRAN JD, GOLOMB HM, LITTLE AG AND WEISCHELBAUM RR. (1988). *Lung Cancer, A Comprehensive Treatise*, pp. 173–241 and 307–397. Grune & Stratton: Orlando, FL, USA.

BROCK I, HIPFNER DR, NIELSEN BS, JENSEN PB, DEELEY RG, COLE SPC AND SEHESTED M. (1995). Sequential coexpression of the multidrug resistance genes MRP and *mdr1* and their products in VP-16 (etoposide)-selected H69 small cell lung cancer cells. *Cancer Res.*, **55**, 459–462.

CHAUDHARY PM AND RONINSON IB. (1993). Induction of multidrug resistance in human cells by transient exposure to different chemotherapeutic drugs. *J. Natl Cancer Inst.*, **85**, 632–639.

CHEN C, CHIN JE, UEDA K, CLARK DP, PASTAN I, GOTTESMAN MM AND RONINSON IB. (1986). Internal duplication and homology with bacterial transport proteins in the *mdr1* (p-glycoprotein) gene from multidrug-resistant human cells. *Cell*, **47**, 381–389.

CHEN C, CLARK D, UEDA K, PASTAN I, GOTTESMAN MM AND RONINSON IB. (1990). Genomic organization of the human multidrug resistance (*MDR1*) gene and origin of P-glycoproteins. *J. Biol Chem.*, **265**, 506–514.

CHEN G, JAFFREZOU J, FLEMING WH, DURAN GE AND SIKIC BI. (1994). Prevalence of multidrug resistance related to activation of the *mdr1* gene in human sarcoma mutants derived by single-step doxorubicin selection. *Cancer Res.*, **54**, 4980–4987.

COLE SPC, BHARDWAJ G, GERLACH JH, MACKIE JE, GRANT CE, ALMQUIST KC, STEWART AJ, KURZ EU, DUNCAN AMV AND DEELEY RG. (1992). Overexpression of a transporter gene in a multidrug-resistant human lung cancer cell line. *Science*, **258**, 1650–1654.

FOJO AT, WHANG-PENG J, GOTTESMAN MM AND PASTAN I. (1985). Amplification of DNA sequences in human multidrug-resistant KB carcinoma cells. *Proc. Natl Acad. Sci. USA*, **82**, 7661–7665.

FOJO AT, UEDA K, SLAMON DJ, POPLACK DG, GOTTESMAN MM AND PASTAN I. (1987). Expression of a multidrug-resistance gene in human tumors and tissues. *Proc. Natl Acad. Sci. USA*, **84**, 265–269.

FRIEDLANDER ML, BELL DR, LEARY J AND DAVEY RA. (1989). Comparison of Western blot analysis and immunohistochemical detection of p-glycoprotein in multidrug resistant cells. *J. Clin. Pathol.*, **42**, 719–722.

GOLDSTEIN LJ, GALSKI H, FOJO A, WILLINGHAM M, LAI S, GAZDAR A, PIRKER R, GREEN A, CRIST W, BRODEUR GM, LIEBER M, COSSMAN J, GOTTESMAN MM AND PASTAN I. (1989). Expression of a multidrug resistance gene in human cancers. *J. Natl Cancer Inst.*, **81,** 116–124.

GROS P, NERIAH YB, CROOP JM AND HOUSMAN DE. (1986). Isolation and expression of a complementary DNA that confers multidrug resistance. *Nature*, **323,** 728–731.

HANSEN HH. (1992). Management of small-cell cancer of the lung. *Lancet*, **339,** 846–849.

HOLZMAYER TA, HILSENBECK S, VON HOFF DDV AND RONINSON IB. (1992). Clinical correlates of *MDR1* (P-glycoprotein) gene expression in ovarian and small-cell lung carcinoma. *J. Natl Cancer Inst.*, **84,** 1486–1491.

INABA M, KOBAYASHI T, TASHIRO T AND SAKURAI Y. (1988). Pharmacokinetic approach to rational therapeutic doses for human tumour-bearing nude mice. *Jpn. J. Cancer Res.*, **79,** 509–516.

INABA M, KOBAYASHI T, TASHIRO T, SAKURAI Y, MARUO K, OHNISHI Y, UEYAMA Y AND NOMURA T. (1989). Evaluation of antitumour activity in a human breast tumour/nude mouse model with a special emphasis on treatment dose. *Cancer*, **64,** 1577–1582.

LAI S, GOLDSTEIN LJ, GOTTESMAN MM, PASTAN I, TSAI C, JOHNSON BE, MULSHINE JL, IHDE DC, KAYSER K AND GAZDER AF. (1989). *MDR1* gene expression in lung cancer. *J. Natl Cancer Inst.*, **81,** 1144–1150.

NAKAGAWA K, SAIJO N, TSUCHIDA S, SAKAI M, TSUNOKAWA Y, YOKOTA J, MURAMATSU M, SATO K, TERADA M AND TEW KD. (1990). Glutathione *S*-transferase $\pi$ as a determinant of drug resistance in transfectant cell lines. *J. Biol. Chem.*, **265,** 4296–4301.

NOONAN KE, BECK C, HOLZMAYER TA, CHIN JE, WUNDER JS, ANDRULIS IL, GAZDAR AF, WILLMAN CL, GRIFFITH B, VON HOFF DD AND RONINSON IB. (1990). Quantitative analysis of *MDR1* (multidrug resistance) gene expression in human tumours by polymerase chain reaction. *Proc. Natl Acad. Sci. USA*, **87,** 7160–7164.

OTA E, ABE Y, OSHIKA Y, OZEKI Y, IWASAKI M, INOUE H, YAMAZAKI H, UEYAMA Y, TAKAGI K, OGATA T, TAMAOKI N AND NAKAMURA M. (1995). Expression of the multidrug resistance-associated protein (*MRP*) gene in non-small cell lung cancer. *Br. J. Cancer*, **72,** 550–554.

RONINSON IB. (1991). *Molecular and Cellular Biology of Multidrug Resistance in Tumour Cells.* pp. 91–104. Plenum Press: New York.

SAMBROOK J, FRITSC E AND MANIATIS T. (1989). *Molecular Cloning: A Laboratory Manual*, 2nd edn, Vol. 1, pp. 7–11. Cold Spring Harbor Laboratory Press: New York.

STARLING JJ, MACIAK RS, HINSON NA, HOSKINS J, LAGUZZA BC, GADSKI RA, STRAND J, RITTMAN-GRAUER L, DERHERDT SV, BUMOL TF AND MOORE RE. (1990). *In vivo* selection of human tumour cells resistant to monoclonal antibody–vinca alkaloid immunoconjugates. *Cancer Res.*, **50,** 7634–7640.

TOTH K, VAUGHAN MM, SLOCUM HK, ARREDONDO MA, TAKITA H, BAKER RM AND RUSTUM YM. (1994). New immunohistochemical 'sandwich' staining method for MDR1 P-glycoprotein detection with JSB-1 monoclonal antibody in formalin-fixed, paraffin-embedded human tissues. *Am. J. Pathol.*, **144,** 227–236.

UEDA K, CLARK DP, CHEN C, RONINSON IB, GOTTESMAN MM AND PASTAN I. (1987). The human multidrug resistance (*mdr1*) gene. *J. Biol. Chem.*, **262,** 505–508.

VERSANTVOORT CHM, BROXTERMAN HJ, PINEDO HM, DE VRIES EGE, FELLER N, KUIPER CM AND LANKELMA J. (1992). Energy-dependent processes involved in reduced drug accumulation in multidrug-resistant human lung cancer cell lines without P-glycoprotein expression. *Cancer Res.*, **52,** 17–23.

VOLM M, MATTERN J AND SAMSEL B. (1991). Overexpression of P-glycoprotein and glutathione *S*-transferase-$\pi$ in resistant non-small cell lung carcinomas of smokers. *Br. J. Cancer*, **64,** 700–704.

WILLIAMS CJ. (1989). Chemotherapy of non-small cell lung cancer. *Br. J. Cancer*, **60,** 9–11.

ZAMAN GJR, VERSANTVOORT CHM, SMIT JJM, EIJDEMS EWHM, DE HAAS M, SMITH AJ, BROXTERMAN HJ, MULDER NH, DE VRIES EGE, BAAS F AND BORST P. (1993). Analysis of the expression of *MRP*, the gene for a new putative transmembrane drug transporter, in human multidrug resistant lung cancer cell lines. *Cancer Res.*, **53,** 1747–1750.

ZWELLING LA, SLOVAK ML, DOROSHOW JH, HINDS M, CHAN D, PARKER E, MAYES J, SIE KL, MELTZER PS AND TRENT JM. (1990). HT1080/DR: A p-glycoprotein-negative human fibrosarcoma cell line exhibiting resistance to topoisomerase II-reactive drugs despite the presence of a drug-sensitive topoisomerase II. *J. Natl Cancer Inst.*, **82,** 1553–1561.